# Supplementary material for: A Critical View of the Application of the APEX Software (Aqueous Photochemistry of Environmentally-Occurring Xenobiotics) to Predict Photoreaction Kinetics in Surface Freshwaters
Source: Molecules. 2019 Dec 18;25(1):9. doi: 10.3390/molecules25010009 (PMC7017383; doi:10.3390/molecules25010009)
Supplement: Supplementary file 1 [file molecules-25-00009-s001.zip › 3.2.4_gcc-4.4.0.zip!/3.2.4_gcc-4.4.0/3.2.4_gcc-4.4.0/doc/octave/html/interpreter/Differential_002dAlgebraic-Equations.html]

Differential-Algebraic Equations - Untitled


Previous: Ordinary Differential Equations,
Up: Differential Equations


---

### 23.2 Differential-Algebraic Equations

The function `daspk` can be used to solve DAEs of the form

```
     0 = f (x-dot, x, t),    x(t=0) = x_0, x-dot(t=0) = x-dot_0
```

where
x-dot
is the derivative of x. The equation is solved using Petzold's
DAE solver Daspk.

— Loadable Function: [x, xdot, istate, msg] = **daspk** (fcn, x\_0, xdot\_0, t, t\_crit)  
> Solve the set of differential-algebraic equations
>
> ```
>           0 = f (x, xdot, t)
> ```
>
> with
>
> ```
>           x(t_0) = x_0, xdot(t_0) = xdot_0
> ```
>
> The solution is returned in the matrices x and xdot,
> with each row in the result matrices corresponding to one of the
> elements in the vector t. The first element of t
> should be t\_0 and correspond to the initial state of the
> system x\_0 and its derivative xdot\_0, so that the first
> row of the output x is x\_0 and the first row
> of the output xdot is xdot\_0.
>
> The first argument, fcn, is a string, inline, or function handle
> that names the function f to call to compute the vector of
> residuals for the set of equations. It must have the form
>
> ```
>           res = f (x, xdot, t)
> ```
>
> in which x, xdot, and res are vectors, and t is a
> scalar.
>
> If fcn is a two-element string array or a two-element cell array
> of strings, inline functions, or function handles, the first element names
> the function f described above, and the second element names a
> function to compute the modified Jacobian
>
> ```
>                 df       df
>           jac = -- + c ------
>                 dx     d xdot
> ```
>
> The modified Jacobian function must have the form
>
> ```
>           jac = j (x, xdot, t, c)
> ```
>
> The second and third arguments to `daspk` specify the initial
> condition of the states and their derivatives, and the fourth argument
> specifies a vector of output times at which the solution is desired,
> including the time corresponding to the initial condition.
>
> The set of initial states and derivatives are not strictly required to
> be consistent. If they are not consistent, you must use the
> `daspk_options` function to provide additional information so
> that `daspk` can compute a consistent starting point.
>
> The fifth argument is optional, and may be used to specify a set of
> times that the DAE solver should not integrate past. It is useful for
> avoiding difficulties with singularities and points where there is a
> discontinuity in the derivative.
>
> After a successful computation, the value of istate will be
> greater than zero (consistent with the Fortran version of Daspk).
>
> If the computation is not successful, the value of istate will be
> less than zero and msg will contain additional information.
>
> You can use the function `daspk_options` to set optional
> parameters for `daspk`.
>
>
>
> **See also:** dassl.

— Loadable Function: **daspk\_options** (opt, val)  
> When called with two arguments, this function
> allows you set options parameters for the function `daspk`.
> Given one argument, `daspk_options` returns the value of the
> corresponding option. If no arguments are supplied, the names of all
> the available options and their current values are displayed.
>
> Options include
>
> `"absolute tolerance"`: Absolute tolerance. May be either vector or scalar. If a vector, it must match the dimension of the state vector, and the relative tolerance must also be a vector of the same length. `"relative tolerance"`: Relative tolerance. May be either vector or scalar. If a vector, it must match the dimension of the state vector, and the absolute tolerance must also be a vector of the same length. The local error test applied at each integration step is ``` abs (local error in x(i)) <= rtol(i) * abs (Y(i)) + atol(i) ``` `"compute consistent initial condition"`: Denoting the differential variables in the state vector by Y\_d and the algebraic variables by Y\_a, `ddaspk` can solve one of two initialization problems: 1. Given Y\_d, calculate Y\_a and Y'\_d- Given Y', calculate Y. In either case, initial values for the given components are input, and initial guesses for the unknown components must also be provided as input. Set this option to 1 to solve the first problem, or 2 to solve the second (the default is 0, so you must provide a set of initial conditions that are consistent). If this option is set to a nonzero value, you must also set the `"algebraic variables"` option to declare which variables in the problem are algebraic. `"use initial condition heuristics"`: Set to a nonzero value to use the initial condition heuristics options described below. `"initial condition heuristics"`: A vector of the following parameters that can be used to control the initial condition calculation. `MXNIT`: Maximum number of Newton iterations (default is 5). `MXNJ`: Maximum number of Jacobian evaluations (default is 6). `MXNH`: Maximum number of values of the artificial stepsize parameter to be tried if the `"compute consistent initial condition"` option has been set to 1 (default is 5). Note that the maximum total number of Newton iterations allowed is `MXNIT*MXNJ*MXNH` if the `"compute consistent initial condition"` option has been set to 1 and `MXNIT*MXNJ` if it is set to 2. `LSOFF`: Set to a nonzero value to disable the linesearch algorithm (default is 0). `STPTOL`: Minimum scaled step in linesearch algorithm (default is eps^(2/3)). `EPINIT`: Swing factor in the Newton iteration convergence test. The test is applied to the residual vector, premultiplied by the approximate Jacobian. For convergence, the weighted RMS norm of this vector (scaled by the error weights) must be less than `EPINIT*EPCON`, where `EPCON` = 0.33 is the analogous test constant used in the time steps. The default is `EPINIT` = 0.01. `"print initial condition info"`: Set this option to a nonzero value to display detailed information about the initial condition calculation (default is 0). `"exclude algebraic variables from error test"`: Set to a nonzero value to exclude algebraic variables from the error test. You must also set the `"algebraic variables"` option to declare which variables in the problem are algebraic (default is 0). `"algebraic variables"`: A vector of the same length as the state vector. A nonzero element indicates that the corresponding element of the state vector is an algebraic variable (i.e., its derivative does not appear explicitly in the equation set. This option is required by the `compute consistent initial condition"` and `"exclude algebraic variables from error test"` options. `"enforce inequality constraints"`: Set to one of the following values to enforce the inequality constraints specified by the `"inequality constraint types"` option (default is 0). 1. To have constraint checking only in the initial condition calculation.- To enforce constraint checking during the integration.- To enforce both options 1 and 2. `"inequality constraint types"`: A vector of the same length as the state specifying the type of inequality constraint. Each element of the vector corresponds to an element of the state and should be assigned one of the following codes -2: Less than zero. -1: Less than or equal to zero. 0: Not constrained. 1: Greater than or equal to zero. 2: Greater than zero. This option only has an effect if the `"enforce inequality constraints"` option is nonzero. `"initial step size"`: Differential-algebraic problems may occasionally suffer from severe scaling difficulties on the first step. If you know a great deal about the scaling of your problem, you can help to alleviate this problem by specifying an initial stepsize (default is computed automatically). `"maximum order"`: Restrict the maximum order of the solution method. This option must be between 1 and 5, inclusive (default is 5). `"maximum step size"`: Setting the maximum stepsize will avoid passing over very large regions (default is not specified).

Octave also includes Dassl, an earlier version of Daspk,
and dasrt, which can be used to solve DAEs with constraints
(stopping conditions).

— Loadable Function: [x, xdot, istate, msg] = **dassl** (fcn, x\_0, xdot\_0, t, t\_crit)  
> Solve the set of differential-algebraic equations
>
> ```
>           0 = f (x, xdot, t)
> ```
>
> with
>
> ```
>           x(t_0) = x_0, xdot(t_0) = xdot_0
> ```
>
> The solution is returned in the matrices x and xdot,
> with each row in the result matrices corresponding to one of the
> elements in the vector t. The first element of t
> should be t\_0 and correspond to the initial state of the
> system x\_0 and its derivative xdot\_0, so that the first
> row of the output x is x\_0 and the first row
> of the output xdot is xdot\_0.
>
> The first argument, fcn, is a string, inline, or function handle
> that names the function f to call to compute the vector of
> residuals for the set of equations. It must have the form
>
> ```
>           res = f (x, xdot, t)
> ```
>
> in which x, xdot, and res are vectors, and t is a
> scalar.
>
> If fcn is a two-element string array or a two-element cell array
> of strings, inline functions, or function handles, the first element names
> the function f described above, and the second element names a
> function to compute the modified Jacobian
>
> ```
>                 df       df
>           jac = -- + c ------
>                 dx     d xdot
> ```
>
> The modified Jacobian function must have the form
>
> ```
>           jac = j (x, xdot, t, c)
> ```
>
> The second and third arguments to `dassl` specify the initial
> condition of the states and their derivatives, and the fourth argument
> specifies a vector of output times at which the solution is desired,
> including the time corresponding to the initial condition.
>
> The set of initial states and derivatives are not strictly required to
> be consistent. In practice, however, Dassl is not very good at
> determining a consistent set for you, so it is best if you ensure that
> the initial values result in the function evaluating to zero.
>
> The fifth argument is optional, and may be used to specify a set of
> times that the DAE solver should not integrate past. It is useful for
> avoiding difficulties with singularities and points where there is a
> discontinuity in the derivative.
>
> After a successful computation, the value of istate will be
> greater than zero (consistent with the Fortran version of Dassl).
>
> If the computation is not successful, the value of istate will be
> less than zero and msg will contain additional information.
>
> You can use the function `dassl_options` to set optional
> parameters for `dassl`.
>
>
>
> **See also:** daspk, dasrt, lsode.

— Loadable Function: **dassl\_options** (opt, val)  
> When called with two arguments, this function
> allows you set options parameters for the function `dassl`.
> Given one argument, `dassl_options` returns the value of the
> corresponding option. If no arguments are supplied, the names of all
> the available options and their current values are displayed.
>
> Options include
>
> `"absolute tolerance"`: Absolute tolerance. May be either vector or scalar. If a vector, it must match the dimension of the state vector, and the relative tolerance must also be a vector of the same length. `"relative tolerance"`: Relative tolerance. May be either vector or scalar. If a vector, it must match the dimension of the state vector, and the absolute tolerance must also be a vector of the same length. The local error test applied at each integration step is ``` abs (local error in x(i)) <= rtol(i) * abs (Y(i)) + atol(i) ``` `"compute consistent initial condition"`: If nonzero, `dassl` will attempt to compute a consistent set of initial conditions. This is generally not reliable, so it is best to provide a consistent set and leave this option set to zero. `"enforce nonnegativity constraints"`: If you know that the solutions to your equations will always be nonnegative, it may help to set this parameter to a nonzero value. However, it is probably best to try leaving this option set to zero first, and only setting it to a nonzero value if that doesn't work very well. `"initial step size"`: Differential-algebraic problems may occasionally suffer from severe scaling difficulties on the first step. If you know a great deal about the scaling of your problem, you can help to alleviate this problem by specifying an initial stepsize. `"maximum order"`: Restrict the maximum order of the solution method. This option must be between 1 and 5, inclusive. `"maximum step size"`: Setting the maximum stepsize will avoid passing over very large regions (default is not specified). `"step limit"`: Maximum number of integration steps to attempt on a single call to the underlying Fortran code.

— Loadable Function: [x, xdot, t\_out, istat, msg] = **dasrt** (fcn [, g], x\_0, xdot\_0, t [, t\_crit])  
> Solve the set of differential-algebraic equations
>
> ```
>           0 = f (x, xdot, t)
> ```
>
> with
>
> ```
>           x(t_0) = x_0, xdot(t_0) = xdot_0
> ```
>
> with functional stopping criteria (root solving).
>
> The solution is returned in the matrices x and xdot,
> with each row in the result matrices corresponding to one of the
> elements in the vector t\_out. The first element of t
> should be t\_0 and correspond to the initial state of the
> system x\_0 and its derivative xdot\_0, so that the first
> row of the output x is x\_0 and the first row
> of the output xdot is xdot\_0.
>
> The vector t provides an upper limit on the length of the
> integration. If the stopping condition is met, the vector
> t\_out will be shorter than t, and the final element of
> t\_out will be the point at which the stopping condition was met,
> and may not correspond to any element of the vector t.
>
> The first argument, fcn, is a string, inline, or function handle
> that names the function f to call to compute the vector of
> residuals for the set of equations. It must have the form
>
> ```
>           res = f (x, xdot, t)
> ```
>
> in which x, xdot, and res are vectors, and t is a
> scalar.
>
> If fcn is a two-element string array or a two-element cell array
> of strings, inline functions, or function handles, the first element names
> the function f described above, and the second element names a
> function to compute the modified Jacobian
>
> ```
>                 df       df
>           jac = -- + c ------
>                 dx     d xdot
> ```
>
> The modified Jacobian function must have the form
>
> ```
>           jac = j (x, xdot, t, c)
> ```
>
> The optional second argument names a function that defines the
> constraint functions whose roots are desired during the integration.
> This function must have the form
>
> ```
>           g_out = g (x, t)
> ```
>
> and return a vector of the constraint function values.
> If the value of any of the constraint functions changes sign, Dasrt
> will attempt to stop the integration at the point of the sign change.
>
> If the name of the constraint function is omitted, `dasrt` solves
> the same problem as `daspk` or `dassl`.
>
> Note that because of numerical errors in the constraint functions
> due to roundoff and integration error, Dasrt may return false
> roots, or return the same root at two or more nearly equal values of
> T. If such false roots are suspected, the user should consider
> smaller error tolerances or higher precision in the evaluation of the
> constraint functions.
>
> If a root of some constraint function defines the end of the problem,
> the input to Dasrt should nevertheless allow integration to a
> point slightly past that root, so that Dasrt can locate the root
> by interpolation.
>
> The third and fourth arguments to `dasrt` specify the initial
> condition of the states and their derivatives, and the fourth argument
> specifies a vector of output times at which the solution is desired,
> including the time corresponding to the initial condition.
>
> The set of initial states and derivatives are not strictly required to
> be consistent. In practice, however, Dassl is not very good at
> determining a consistent set for you, so it is best if you ensure that
> the initial values result in the function evaluating to zero.
>
> The sixth argument is optional, and may be used to specify a set of
> times that the DAE solver should not integrate past. It is useful for
> avoiding difficulties with singularities and points where there is a
> discontinuity in the derivative.
>
> After a successful computation, the value of istate will be
> greater than zero (consistent with the Fortran version of Dassl).
>
> If the computation is not successful, the value of istate will be
> less than zero and msg will contain additional information.
>
> You can use the function `dasrt_options` to set optional
> parameters for `dasrt`.
>
>
>
> **See also:** daspk, dasrt, lsode.

— Loadable Function: **dasrt\_options** (opt, val)  
> When called with two arguments, this function
> allows you set options parameters for the function `dasrt`.
> Given one argument, `dasrt_options` returns the value of the
> corresponding option. If no arguments are supplied, the names of all
> the available options and their current values are displayed.
>
> Options include
>
> `"absolute tolerance"`: Absolute tolerance. May be either vector or scalar. If a vector, it must match the dimension of the state vector, and the relative tolerance must also be a vector of the same length. `"relative tolerance"`: Relative tolerance. May be either vector or scalar. If a vector, it must match the dimension of the state vector, and the absolute tolerance must also be a vector of the same length. The local error test applied at each integration step is ``` abs (local error in x(i)) <= ... rtol(i) * abs (Y(i)) + atol(i) ``` `"initial step size"`: Differential-algebraic problems may occasionally suffer from severe scaling difficulties on the first step. If you know a great deal about the scaling of your problem, you can help to alleviate this problem by specifying an initial stepsize. `"maximum order"`: Restrict the maximum order of the solution method. This option must be between 1 and 5, inclusive. `"maximum step size"`: Setting the maximum stepsize will avoid passing over very large regions. `"step limit"`: Maximum number of integration steps to attempt on a single call to the underlying Fortran code.

See K. E. Brenan, et al., Numerical Solution of Initial-Value
Problems in Differential-Algebraic Equations, North-Holland (1989) for
more information about the implementation of Dassl.
